# Supplementary material for: Evaluation of a health promotion intervention associated with birthing centres in rural Nepal
Source: PLoS One. 2020 May 22;15(5):e0233607. doi: 10.1371/journal.pone.0233607 (PMC7244127; doi:10.1371/journal.pone.0233607)
Supplement: S1 Table — (DOCX) [file pone.0233607.s001.docx]

**S1 Table: Association of intervention with other variables**

| Age of women during study | | Intervention | | p-value | Cramer’s V value |
| --- | --- | --- | --- | --- | --- |
|  |  | **Pre** | **Post** |  |  |
| Women’s education  Illiterate (N, %)  Adjusted Residual | 270/407 (66.3)  12.0 | | 205/699(29.3)  -12.0 | <.001* | .362 |
| Choice of birthplace  Primary care facilities (N, %)  Adjusted residuals | | 49/420 (11.7)  -10.3 | 286/699 (40.9)  10.3 | <.001* | .343 |
| Decision maker for birthplace  Woman and family (N, %)  Adjusted residuals | | 13/420 (3.1)  -18.2 | 401/699 (57.4)  18.2 | <.001* | .569 |
| Satisfaction with delivery service  Highly satisfied (N, %)  Adjusted Residual | | 296/420 (70.5)  -12.8 | 678/699 (97.0)  12.8 | <.001* | .387 |
