# Supplementary material for: Evaluation of a health promotion intervention associated with birthing centres in rural Nepal
Source: PLoS One. 2020 May 22;15(5):e0233607. doi: 10.1371/journal.pone.0233607 (PMC7244127; doi:10.1371/journal.pone.0233607)
Supplement: Attachment — Submitted filename: Response to reviewers.docx [file pone.0233607.s003.docx]

Abstract

- Please add numeric results to the results section

Thank you for your comment, we have added numeric data to the results section of the abstract.

-Avoid stating “significantly more likely”. The post-intervention survey provided evidence that women were more likely to … than prior to the intervention.

Thank you for the suggestion. We have used following sentences in the results section of abstract “The post-intervention survey provided evidence that women were more likely to give birth at primary care facilities (OR 5.60, p-value <0.001) than prior to the intervention. Similarly, the likelihood of giving birth at a health facility increased if decision for birthplace was made jointly by women and family members for primary care facilities (OR 1.76, p-value 0.023) and hospitals/tertiary care facilities (OR 1.78, p-value 0.020). If women had less than four ANC visits, then they were less likely to give birth at primary care facilities (OR 0.39, p-value <0.001) or hospitals/tertiary care facilities (OR 0.63, p-value 0.014). Finally, women were less likely to give birth at primary care facilities if they had only primary level of education (OR 0.49, p-value 0.014)”

In the tracked copy, P5. Line 73. Need to start a sentence with a word, not an abbreviation.

We have done this.

Table 3. This needs the number and percent for each response. Some of these are already in Table 1. However, important factors such as knowledge about abortion and money received for childbirth are not really clear. I agree with Reviewer 1 the reference for each of these needs to be “No”, rather than “Don’t know”. It is possible to rearrange the data in the statistical package to do this. I also don’t understand why the emphasis is placed on being “somewhat satisfied” or “highly dissatisfied” with childbirth – I would have thought the hope was that the intervention increased the proportion of women who were positive about the experience, rather than emphasising dissatisfaction.  I also presume the asterisks indicate the level of significance. These are not needed, as the reader can see this from the p=values – the reader also needs to focus on the width of the confidence intervals, which are more meaningful than a p-value.

Thank you for this important suggestion. As you have mentioned, we have not included the number and percent for each response in Table 3, since it is already mentioned in Table 1 and 2 and it would not be possible to include this Table 3 due to space constraints. Your comments about using ‘don’t know’ as reference category are valid, and we have rearranged the data in the SPSS for ‘knowledge about abortion’ and ‘money received for childbirth’. The reference category for these variables now are ‘No’ rather than ‘Don’t know’. We had to conduct the regression analysis again, therefore there is slight change in the figures in Table 3. The categories for ‘satisfaction with childbirth’ was based on the pre-intervention survey questionnaire and was not revised for the post-intervention survey. We understand that including this in the regression analysis does not clarify anything, so we chose not to include this in this analysis.

As per your suggestion we have removed the asterisk sign as the confidence interval does tell this.

The supplementary table was not available for view in this revision. Please provide it again.

We have provided this again.
